# Dynamic changes in the hippocampal neuronal circuits activity following acute stress revealed by miniature fluorescence microscopy imaging

**DOI:** 10.1186/s13041-024-01168-5

**Published:** 2024-12-18

**Authors:** Evgenii Gerasimov, Ekaterina Pchitskaya, Olga Vlasova, Ilya Bezprozvanny

**Affiliations:** Laboratory of Molecular Neurodegeneration, Peter the Great St. Petersburg Polytechnic University, Polytechnicheskaya St. 29, 195220 St. Petersburg, Russia

**Keywords:** Miniature fluorescence microscopy, Miniscope, Hippocampus, Neuronal circuits, Acute stress, Quantitative analysis, Homeostatic stability, Calcium imaging

## Abstract

Coordinated activity of neuronal ensembles is a basis for information processing in the brain. Recent development of miniscope imaging technology enabled recordings of neuronal circuits activity in vivo in freely behaving animals. Acute stress is believed to affect various hippocampal functions, especially memory. In the current study, we utilized miniscope imaging to investigate the hippocampal neuronal circuits properties in a mouse as function of time and immediately in response to an acute stress, induced by passive restraint, 3 h and 10 days after. Comprehensive quantitative analysis of network activity changes at the neuronal ensembles level revealed highly stable neuronal activity parameters, which exhibited a rapid and robust shift in response to acute stress stimulation. This shift was accompanied by the restructuring of the pairwise-correlated neuronal pairs. Remarkably, we discovered that ensembles activity characteristics returned to the initial state following recovery period, demonstrating hippocampal homeostatic stability at the neuronal circuits level. Obtained results provide an evidence about hippocampal neuronal ensembles activity in response to acute stress over time.

**Supplementary Information:**

The online version contains supplementary material available at 10.1186/s13041-024-01168-5.

## Introduction

Miniature fluorescence microscopy is a powerful tool in the arsenal of modern neuroscience. This technique allows researchers to monitor the activities of hundreds of neurons simultaneously in freely behaving mice [1–5]. The miniscope method is advantageous because it permits unrestricted movement of laboratory mice due to its small weight approximately 3 g. The visualization of neuronal excitation is achieved by expressing in the neurons calcium-sensitive indicators such as GCaMPs [6–8]. These indicators increase fluorescence intensity in response to elevations in intracellular calcium concentrations, an indirect correlate of neuronal excitation.

The hippocampal neuronal network plays an indispensable role in higher cognitive functions, including the processes of learning [9–11], memory formation [12–15] and recall [16]. Many studies of hippocampal neuronal activity in vivo are dedicated to understanding spatial memory formation [17, 18] and investigating place cell activity [5, 19–22]. In common, these studies are based on some distinct cells activity analysis connected to place determination, environmental changes or memory investigation. However, it is quite unclear whether the overall activity of the certain brain’s region neuronal circuits maintains homeostasis or exhibits variability day to day. Is it possible to elucidate and characterize the behavior of the neuronal circuits under identical conditions and discern if it returns to its initial state after a great external shift? To address these questions, we leveraged miniscope imaging technique for neuronal ensembles in vivo recording in dorsal hippocampal area of mice.

The current investigation aims to scrutinize dynamic changes in hippocampal neuronal ensembles activity and the architecture of its neuronal connections after great external perturbation. Changes in neuronal ensemble properties after exposure to strong external stimuli, such as acute stress, are of great interest, as they are believed to promote rapid and prolonged changes in the entire hippocampal structure [23]. It has been recently shown that in the hippocampus acute stress triggers rapid response in protein phosphorylation, gene transcription and protein synthesis [24, 25], has an effect on long-term potentiation [26], alters mouse behavior and spatial memory retrieval [27, 28]. Moreover, acute stress affects various hippocampal-cortex pathways [29], directly impacts the excitability of dorsal hippocampal CA1 neurons [30] and led to significant changes in dorsal hippocampal area obtained via EEG method [31]. However, the direct effect of acute stress on the neuronal ensembles activity in vivo is still being studied. Miniscope imaging technique allowed us to monitor the same neuronal ensembles over days, allowing us to examine the activity and connections of the same neurons over time under normal conditions and after acute stress modelling as a model of the great external exposure. Therefore, current research may shed light on the rapid and long-term changes in the functioning neuronal circuits in the dorsal hippocampus after acute stress modeling.

In this manuscript, it is shown how the hippocampal neuronal ensembles state changes or remains stable over consecutive days as well as after significant external stimuli. Quantitative analysis of the miniscope data was executed using a self-developed toolbox “NeuroActivityToolkit” [32]. Acute stress in mice was induced by 30 min passive restraint [26, 33]. This straightforward method allowed us to examine hippocampal neuronal ensembles reaction to a great external stimulus over a time. Despite the initial severe alterations in various descriptive parameters (activations per minute, network spike degree etc.) detected immediately after acute stress modeling and at a 3-h time-point, the hippocampal neuronal circuits demonstrated remarkable stability and eventually reverted to its initial state on the 10th day after.

## Results

### Number of neuronal activations in CA1 hippocampal neurons is stable over time and is affected by acute stress

**Fig. 1:**
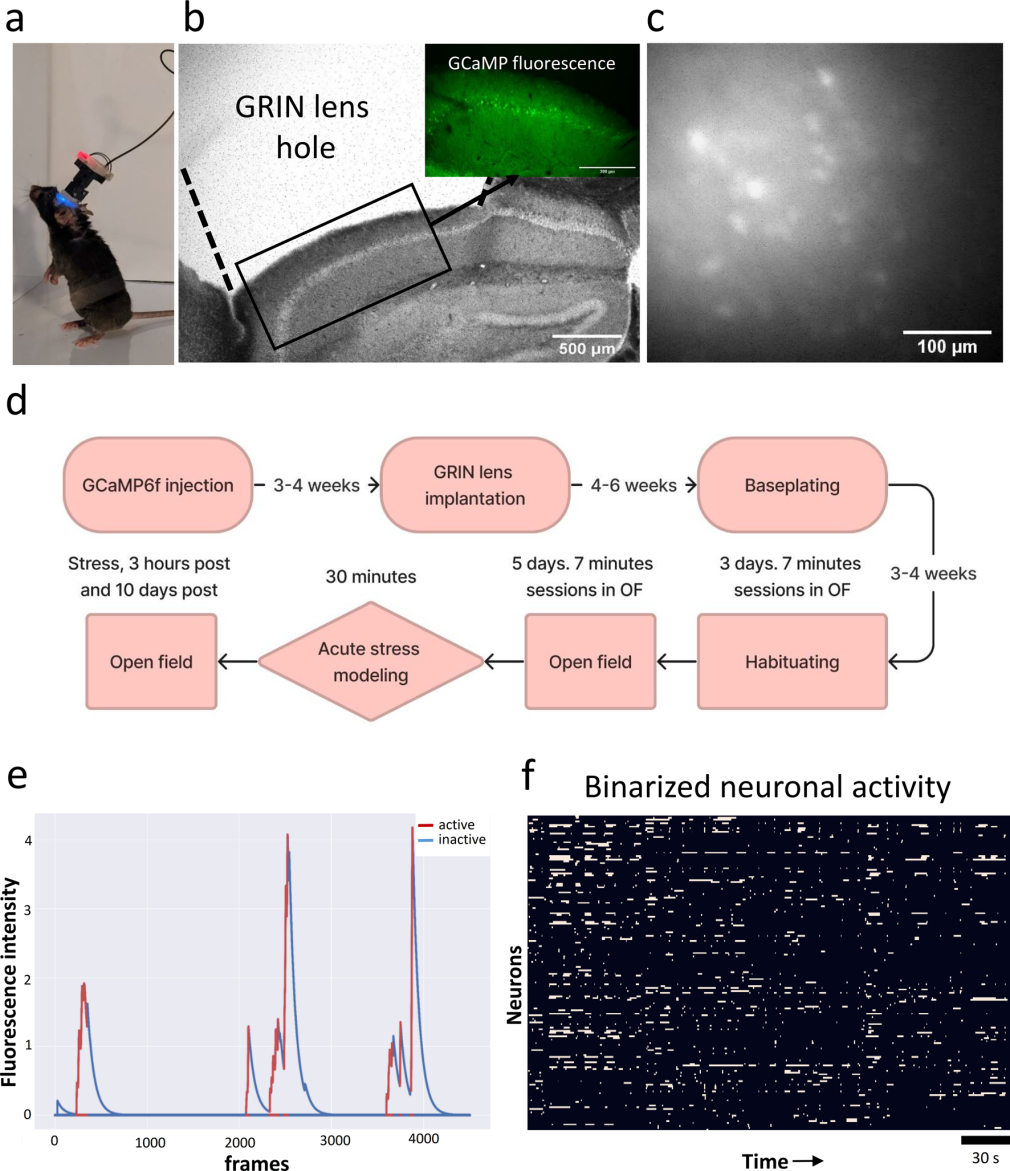
Experimental pipeline schematic presentation. **a** Freely behaving mouse with an attached miniscope v3. **b** Fixed sagittal brain slice with hole above the hippocampus after surgery, 4× magnification (in the right corner fluorescent image of the GCaMP6f fluorescence, 10× magnification). By dotted line GRIN lens boarders are drawn. **c** CA1 hippocampal neurons activity recorded by the miniscope. Illustration represents sum of 1000 frames from a single recording. **d** Timescale for neuronal activity visualization by miniscope in the Open-field behavioral test with acute stress modeling as a great external stimulus. **e** Extracted single neuron activity as active and inactive state using spike method with values “warm” of 50 and “cold” of 0 in “NeuroActivityToolkit”. **f** Recorded neuronal ensembles activity representation in a binarized form for a single recording (mouse 1, baseline day 1, 182 neurons). White lines indicate single neuron activation

To assess the stability properties of the hippocampal neuronal ensembles under both normal conditions and after external stimulus, a miniature fluorescent microscopy method was performed on the 8 month old C57BL/6J mice line (Fig. 1a). To record activity of excitatory hippocampal neurons genetically encoded calcium-sensitive fluorescent indicator GCaMP6f was used (Fig. 1b, c). Miniscope method allows us to record hundreds of neurons over days with stable field of view (Fig. S1) [34]. The CA1 hippocampal neurons state was recorded in freely moving sessions once a day for five consecutive days, serving as a baseline reference. In the current manuscript, we used terms “neuronal circuits” or “neuronal ensembles” for the identification of the group of CA1 neuronal cells that were imaged with the miniscope. To induce an external shift of hippocampal activity, the mice underwent a 30-min acute stress modeling. Then the neuronal circuits activity was recorded immediately after the stress test (referred to as “stress” in all graphs), as well as 3 h (“3 h”) and 10 days (“10 days”). The overall timeline is presented in Fig. 1d. All the data were processed using the “spike” method (Fig. 1e), where only the rapid phase of the calcium indicator signal growth was considered unless otherwise specified. For analysis we have transformed all the neuronal activity into binarized activity (Fig. 1f) [32].

**Fig. 2:**
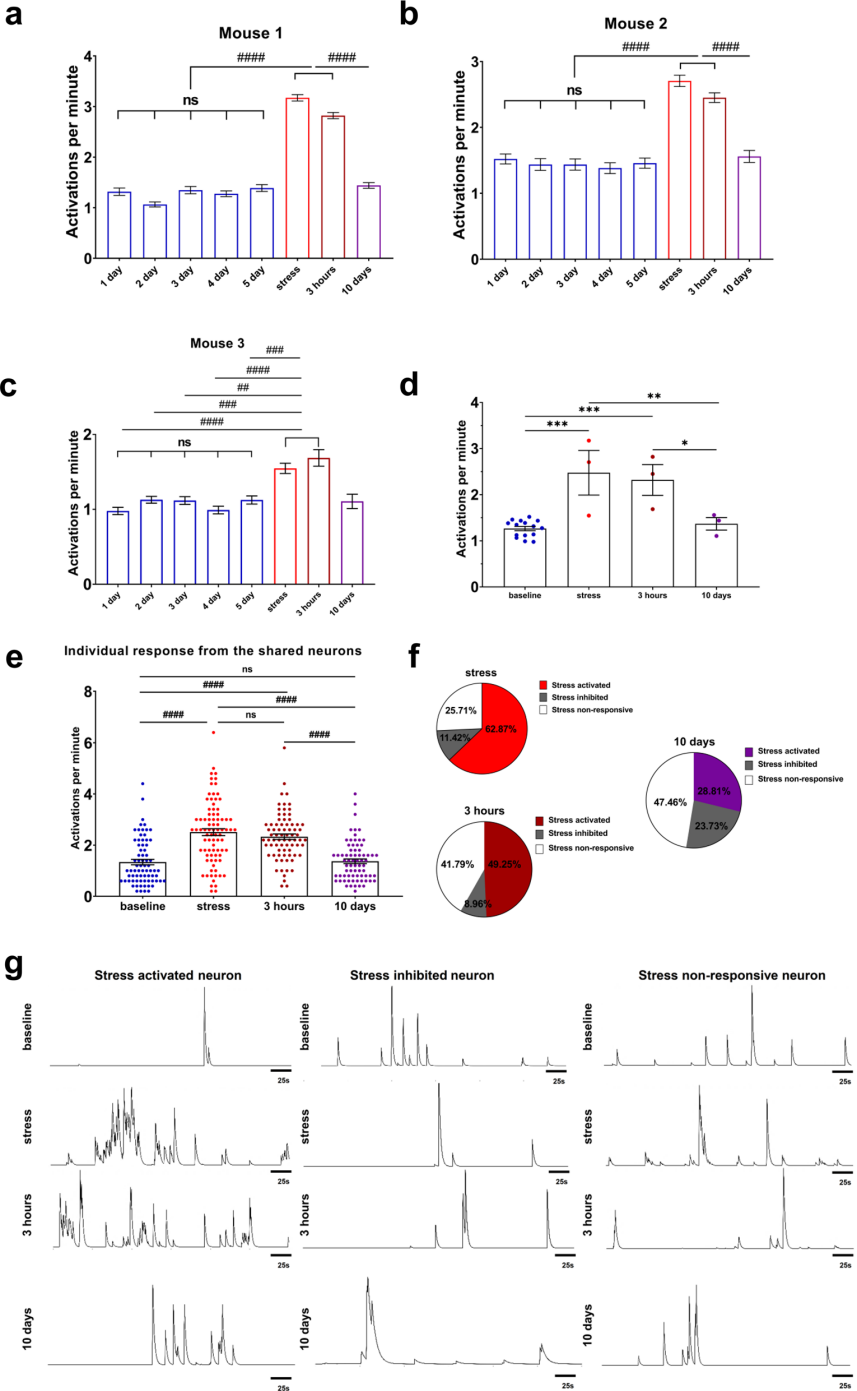
Shift in the activity properties at the single neuronal level as well as in the entire neuronal ensembles after acute stress modeling. **a–c** Calcium events number per minute for individual mouse for single-cell activity comparison. **d** Mean value of calcium events as total neuronal circuits characteristic for all states (baseline (n = 15) vs stress (n = 3), *p* = 0.0002; baseline (n = 15) vs 3 h (n = 3), *p* = 0.0009; baseline (n = 15) vs 10 days (n = 3), *p* = 0.9690; stress (n = 3) vs 3 h (n = 3), *p* = 0.9467; stress (n = 3) vs 10 days (n = 3), *p* = 0.0066; 3 h (n = 3) vs 10 days (n = 3), *p* = 0.0212; Ordinary One-way ANOVA with Tukey test for multiple comparison, F(3,20) = 14.29). **e** Individual response from the shared neurons between sessions for all states. All comparisons are significantly different with *p* < 0.0001, except: stress (n = 73 neurons) vs 3 h (n = 64 neurons), *p* > 0.9999 and baseline (73 neurons) vs 10 days (64 neurons), *p* > 0.9999. **f** Percentage of neurons that responded to stress or not for stress, 3 h and 10 days states. (**g**) Left: representative calcium traces of the same stress activated neuron on day 5 (baseline), stress, 3 h and 10 days states. Middle: representative calcium traces of the same stress inhibited neuron on day 5 (baseline), stress, 3 h and 10 days states Right: representative calcium traces of the same stress non-responsive neuron on day 5 (baseline), stress, 3 h and 10 days states. Scale bars corresponds to 25 s. Kruskal–Wallis test with Dunn’s test for multiple comparisons was used for comparisons in **a**–**e**, ns: no significant difference, #: *p* < 0.05; ##: *p* < 0.01; ###: *p* < 0.001; ####: *p* < 0.0001. Ordinary one-way ANOVA following Tukey post-hoc test was implemented for comparison in **d**, *: *p* < 0.05; **: *p* < 0.01; ***: *p* < 0.001; ****: *p* < 0.0001. All the data presented as mean ± SEM

To validate this statement and confirm whether neuronal activity returns to a stable original point after recovery, we analyzed the mean values of individual neuronal activations. In this case, the overall activity of the neuronal circuits is characterized by the mean level of all neuron’s activity in this network. It can be clearly observed that right after stress and at the 3-h post time point the hippocampal ensembles are in an excited state since the number of activations per minute is elevated (1.27 ± 0.05, n = 15 (baseline) vs 2.48 ± 0.48, n = 3 (stress), *p* = 0.0002; 1.27 ± 0.05, n = 15 (baseline) and 2.32 ± 0.33, n = 3 (3 h), *p* = 0.0009, F(3,20) = 14.29, One-way ANOVA with following Tukey post-hoc test). Moreover, by day 10, the activity of the hippocampal neurons returned to its initial value with no significant differences observed with baseline values (1.27 ± 0.05 (baseline) and 1.36 ± 0.13 (10 days), *p* = 0.9690, F(3,20) = 14.29, One-way ANOVA with following Tukey post-hoc test) (Fig. 2d). For a more detailed visualization, binarized activity of the neurons in all states can be found in Fig. S2(a). Considering the natural variability among individual mice, we have also performed normalized per-mouse analysis of activations per minute (Fig. S3), where the similar changes are stated. Further, to examine neuronal activations at the single-neuron level, we tracked the activity of the neurons shared between the baseline level (day 5) and across stress, 3 h and 10 days states. We have determined significantly increased activity of the same tracked neurons between sessions, consistent with the overall circuits activation as shown above. A great elevation of neuronal activations was observed during the stress state (1.34 ± 0.11, n = 73 neurons (baseline) vs 2.51 ± 0.14, n = 73 neurons (stress), *p* < 0.0001) and at 3 h state (1.34 ± 0.11, n = 73 neurons (baseline) vs 2.33 ± 0.11, n = 64 neurons (3 h), *p* < 0.0001). Moreover, no significant changes were observed 10 days post stress modeling (1.34 ± 0.11, n = 73 neurons (baseline) vs 1.37 ± 0.09, n = 64 neurons (10 days), *p* > 0.9999) (Fig. 2g). While addressing normalized individual response from the shared neurons between sessions, the same tendency was observed (Fig. S3(b)). To validate how acute stress modeling influenced hippocampal neurons, we have analyzed neuronal activations changes in all states. Firstly, we have identified threshold levels for “stress activated” and “stress inhibited” neurons (see Materials and Methods section). Then, activity of the same neurons for stress, 3 h and 10 days states was normalized on the baseline level. We have found that 62.87% of the shared neurons were stress activated, 11.42% stress inhibited and 25.71% did not respond to stress. At the same time, at 3 h mark stress activated neurons relative amount was 49.25, 8.96% were stress inhibited and 41.79% of neurons were stress non-responsive (Fig. 2f). 10 days after great external stimuli, we have identified, that only 28.81% of neurons were stress activated, while activity of 23.73% neurons was inhibited and 47.46% did not respond to stress. Representative calcium traces of the same stress activated, stress inhibited and stress-non responsive neurons across sessions can be found in Fig. 2g.

In conclusion, the averaged neuronal activity in the recorded hippocampal area can be stated as a stable value over days that reverts to its “home” state after reaction to a crucial external perturbation.

### External stimulus induced by acute stress lead to severe changes in the hippocampal neuronal ensembles activity properties

**Fig. 3:**
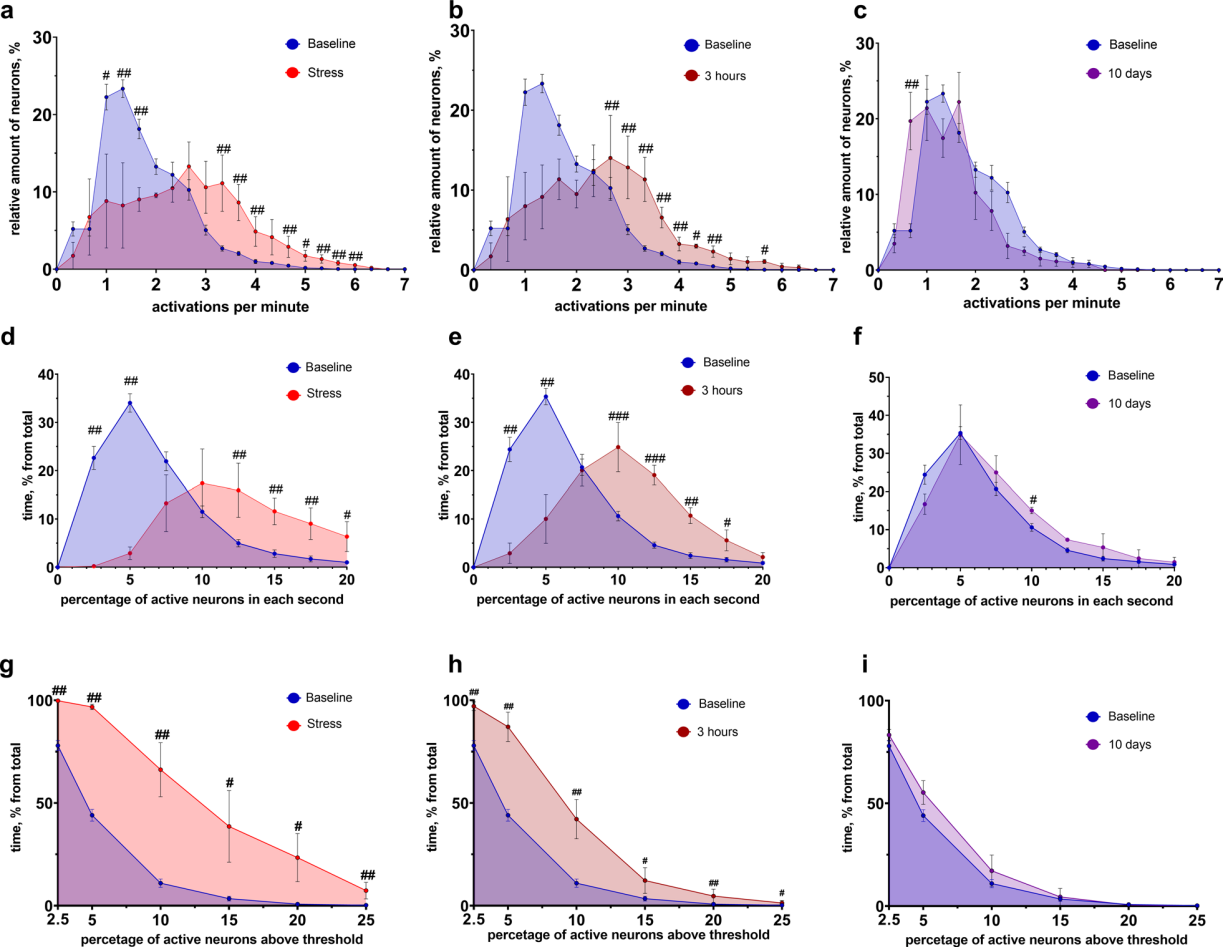
Hippocampal neuronal ensembles activation properties in normal and perturbed conditions. Distributions of neurons percent with given number of activation in normal condition and **a** right after stress (baseline (n = 15) vs stress (n = 3): 0.66–1.00: *p* = 0.0403; 1.00–1.33: *p* = 0.0059; 1.33–1.66: *p* = 0.0029; 3.00–3.33: *p* = 0.0206; 3.33–3.66: *p* = 0.0029; 3.66–4.00: *p* = 0.0250; 4.33–4.66: *p* = 0.0235; 4.66–5.00: *p* = 0.0029; 5.00–5.33: *p* = 0.0103; 5.33–5.66: *p* = 0.0029; 5.66–6.00: *p* = 0.0015, Mann–Whitney test for all). **b** 3 h post acute stress (baseline (n = 15) vs 3 h (n = 3): 2.33–2.66: *p* = 0.0029; 2.66–3.00: *p* = 0.0029; 3.00–3.33: *p* = 0.0029; 3.33–3.66: *p* = 0.0029; 3.66–4.00: *p* = 0.0029; 4.00–4.33: *p* = 0.0015; 4.33–4.66: *p* = 0.0235; 4.66–5.00: *p* = 0.0029; 5.00–5.33: *p* = 0.0103; 5.33–5.66: *p* = 0.0059, Mann–Whitney test for all). **c** 10 days post acute stress (baseline (n = 15) vs 10 days (n = 3): 0.33–0.66: *p* = 0.0208; others are non-significant, Mann–Whitney test for all). Distributions of network spike rate metric in normal condition and **d** right after stress (baseline (n = 15) vs stress (n = 3): 0.0–2.5: *p* = 0.0018; 2.5–5.0: *p* = 0.0018; 10.0–12.5: *p* = 0.0044; 12.5–15.0: *p* = 0.0026; 15.0–17.5: *p* = 0.0044; 17.5–20.0: *p* = 0.0184, Mann–Whitney test for all). **e** 3 h post acute stress (baseline (n = 15) vs 3 h (n = 3): 0.0–2.5: *p* = 0.0018; 2.5–5.0: *p* = 0.0026; 7.5–10.0: *p* = 0.0035; 10.0–12.5: *p* = 0.0009; 12.5–15.0: *p* = 0.0026; 15.0–17.5: *p* = 0.0219, Mann–Whitney test for all). **f** 10 days post acute stress (baseline (n = 15) vs 10 days (n = 3): 7.5–10.0: *p* = 0.0228, others are not significantly differing, Mann–Whitney test for all). Distributions of active cells percentage above threshold in normal condition and **g** right after stress (baseline (n = 15) vs stress (n = 3): 2.5%: *p* = 0.0025; 5.0%: *p* = 0.0012; 10.0%: *p* = 0.0257; 15.0%: *p* = 0.0282; 20.0%: *p* = 0.0221; 25.0%: *p* = 0.0025, Mann–Whitney test for all). **h** 3 h post acute stress (2.5%: *p* = 0.0025; 5.0%: *p* = 0.00025; 10.0%: *p* = 0.0025; 15.0%: *p* = 0.051; 20.0%: *p* = 0.0147; 25.0%: *p* = 0.051, Mann–Whitney test for all). **i** 10 days post acute stress (all the values are not significantly differing, Mann–Whitney test for all). #: *p* < 0.05; ##: *p* < 0.01; ###: *p* < 0.001. All the data presented as mean ± SEM

To investigate dynamic shifts in the hippocampal neuronal network activity, we analyzed metrics related to neuronal activation characteristics. Our first focus was on comparing the distribution of neurons with the given number of activations to observe how strong was the total response of the circuits to the applied stimulus (referred to as the “burst rate”). We compare the distributions for baseline, “stress”, “3 h” and “10 days” which are presented in Fig. 3a–c. These comparisons indicated a noticeable increase in the number of neurons with a larger number of the calcium activations per minute, expressed in the difference in most data points under stressed conditions (Fig. 3a, b). Peak values for the baseline level ranged from 0.66 to 1.00 activation per minute, whereas for stressed conditions they reached 3.00–3.33 for the “stress” state (in comparison with baseline value: n = 15 for baseline and n = 3 for stress, *p* = 0.0206, Mann–Whitney test) and 2.66–3.00 (in comparison with baseline value: n = 15 for baseline and n = 3 for the “3 h”, *p* = 0.0029, Mann–Whitney test) for 3-h post stress time-point. At the same time, the distributions for baseline and 10 days post acute stress modeling were similar (Fig. 3c).

Next, we examined the network spike rate, which represents the proportion of neurons in an active state within distinct time intervals (all recordings were divided into 1-s sections, and the number of active neurons was computed for each interval). These results are depicted in Fig. 3d–f. There was an explicitly expressed shift towards higher values of simultaneously active neurons number after acute stress modeling (Fig. 3d, e), with significant differences between the prevailing amount of the graph points, mirroring the pattern seen in the burst rate metric. For the baseline peak values ranged from 2.5 to 5.0% of active neurons, while for the “stress” state, they reached 10.0–12.5% (compared to baseline: n = 15 for baseline and n = 3 for “stress”, *p* = 0.0044, Mann–Whitney test) and 7.5–10.0% for the “3 h” state (compared to baseline: n = 15 for baseline and n = 3 for “3 h”, *p* = 0.0009, Mann–Whitney test). Nonetheless, on the 10th day, the distributions were mostly similar. Additionally, we examined the network spike duration metric – the time during which the count of concurrently active cells exceeds a predetermined threshold level (Fig. 3g–i). A great elevation of time duration when percent of active neurons was above preset level was registered in the “stress” and “3 h” states. However, on the 10th day, the distribution is also absolutely similar without any significant differences between the same threshold points when compared to the baseline. The same pattern of significantly enlarged network spike duration in stressed conditions could be also found there. Multiple comparison analysis of the presented data can be found in Fig. S4, thus all groups distribution differences are accounted. Moreover, for more precise distribution examination, we have performed cumulative frequency comparison between different states of the neuronal ensembles, where the same changes as observed above are found (Fig. S5).

In summary, the analysis of the activity properties of hippocampal neuronal circuits indicates that acute stress causes significant changes in the activation parameters of hippocampal neurons, with complete recovery after 10 days of rest.

### The amount of weakly-correlated hippocampal neuronal pairs is increased in response to acute stress

Neuronal ensembles work as correlated groups of connected neurons that reflect the processes occurring within the defined brain region [35–37]. This chapter is dedicated to estimation dynamics in pairwise neuronal correlations under normal conditions and after mice exposure to acute stress for shifting the hippocampal neuronal network state from its initial point. To accurately validate changes in the pairwise connections among co-active neurons, Pearson’s correlation coefficient was used. Various approaches of its calculation according to miniscope data can be found here [32].

**Fig. 4:**
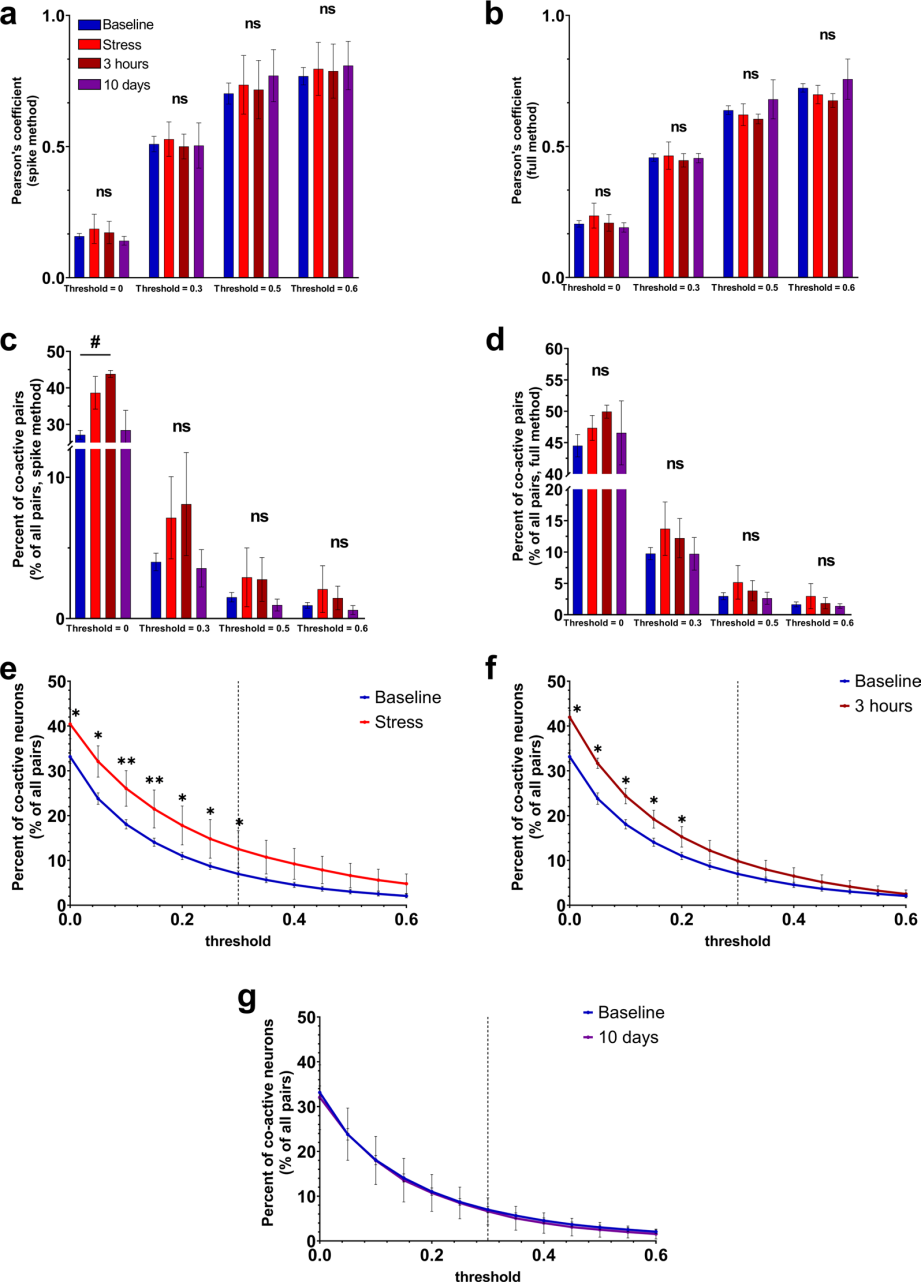
Pairwise correlation properties stay stable while percent of co-active neurons elevates after acute stress. **a** Pearson’s correlation coefficient value for all states with preset threshold values calculated by “spike” method. **b** Pearson’s correlation coefficient value for all states with preset threshold values calculated by “full” method. **c** Number of connected pairs of neurons in relation to all the pairs with preset threshold (threshold = 0; baseline (n = 15) vs 3 h (n = 3): *p* = 0.012, Kruskal–Wallis test following Dunn’s test for multiple comparison). **d** Number of connected pairs of neurons as a fraction from all pairs with preset threshold. Network degree (**e**) right after stress (baseline (n = 15) vs stress (n = 3), threshold: 0.00: *p* = 0.0473; 0.05: *p* = 0.0156; 0.10: *p* = 0.0091; 0.15: *p* = 0.0098; 0.20: *p* = 0.0118; 0.25: *p* = 0.0158; 0.30: *p* = 0.0175; Student’s t-test for all). **f** 3 h post acute stress (baseline (n = 15) vs 3 h (n = 3), threshold: 0.00: *p* = 0.0155; 0.05: *p* = 0.0132; 0.10: *p* = 0.0167; 0.15: *p* = 0.0277; 0.20: *p* = 0.0460; 0.25: *p* = 0.0688; 0.30: *p* = 0.100, Student’s t-test for all). **g** 10 days post acute stress (all differences are non-significant, Student’s t-test). ns-non-significant; #: *p* < 0.05; #: *: *p* < 0.05; **: *p* < 0.01. All the data presented as mean ± SEM. For **e**,** f** and **g** dotted line represents threshold level for “strongly” correlated units

Across various conditions, including non-stressed baseline state, “stress”, “3 h” and “10 days” time-points, Pearson’s coefficient value above a predetermined threshold did not alter in any way (for all of the comparisons presented in Fig. 4a, b: *p* > 0.5035, Kruskal–Wallis test with Dunn’s correction for multiple comparisons). This observation suggests that the increase in neuronal excitation, as evidenced by the elevation of calcium transients number described above, occurred, mostly, in a random manner. Interestingly, the number of strongly connected neurons didn’t vary at all (above the reasonably high value of correlation coefficient of 0.3) between different states of the hippocampal neuronal network as shown in Fig. 4c, d, f, g. The threshold for “strongly” correlated neurons 0.3 was determined as a mean value of 95th percentile of Pearson’s correlation coefficient for all baseline recordings and shifted towards the closest binarized threshold value (see Fig. 4e–g).

However, when comparing the number of co-active neuronal pairs above the preset threshold, determined by Pearson`s correlation coefficient (Fig. 4e–g), significant differences were observed, particularly for threshold values below 0.3. Such elevation in the number of weakly correlated neuronal pairs might play an important role in the total neuronal network representation being a precise way for fine-tuning of the brain region activity and various processes to receiving stimulus [38].

### Redistribution in space of correlated hippocampal neuronal pairs in respond to acute stress

**Fig. 5:**
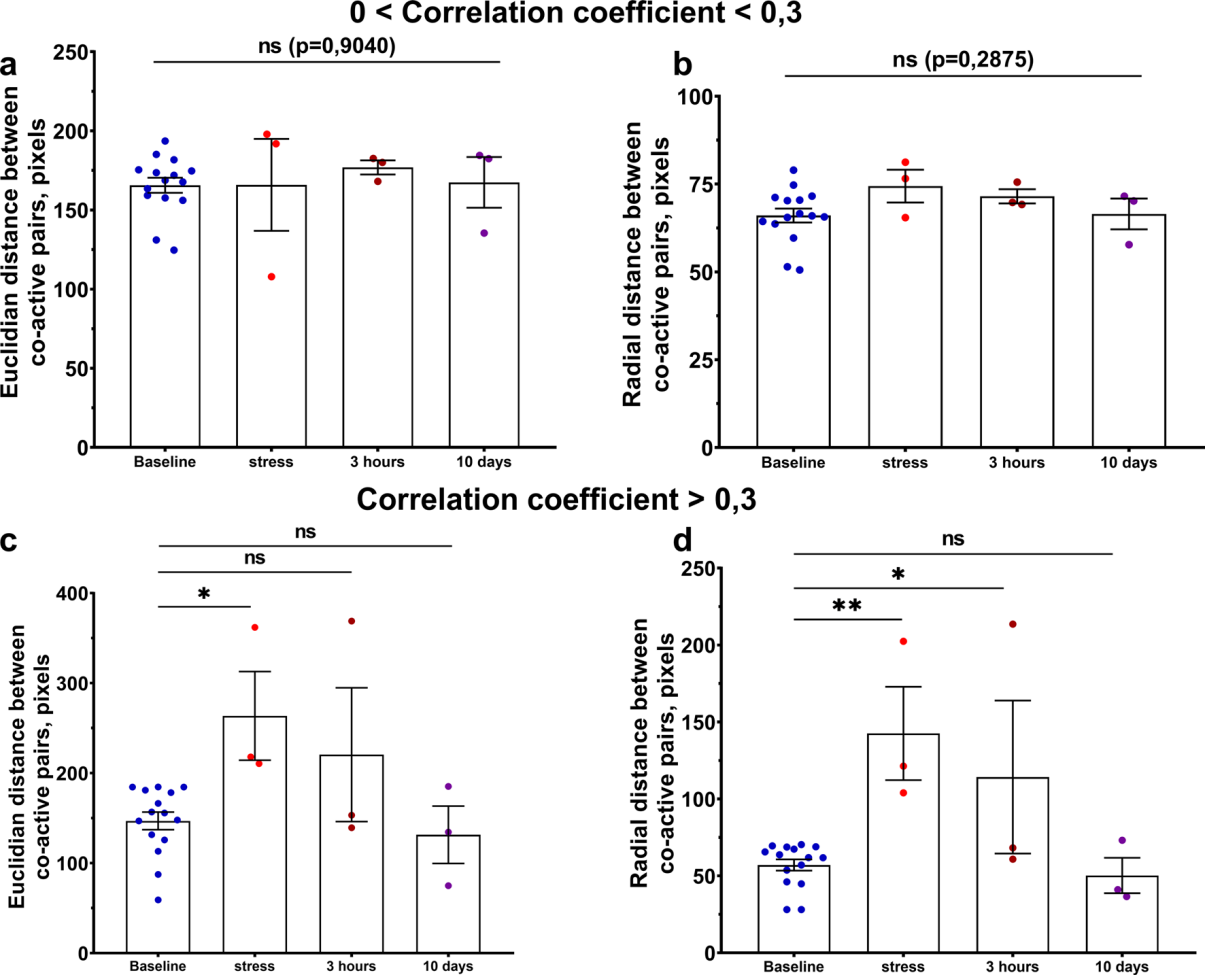
Redistribution of strongly correlated hippocampal neuronal pairs in space in a response to acute stress. **a** Mean Euclidian distance for weakly correlated neurons (*p* = 0.9040, F(3,20) = 0.1869, One-way ANOVA test). **b** Mean radial distance for weakly correlated neurons (*p* = 0.2875, F(3,20) = 1.347, One-way ANOVA test). **c** Mean Euclidian distance for strongly correlated neurons (baseline (n = 15) vs stress (n = 3): *p* = 0.0190, F(3,20) = 4.178, One-way ANOVA with Dunnett’s post-hoc test). **d** Mean radial distance between strongly correlated neuronal pairs (baseline (n = 15) vs stress (n = 3): *p* = 0.0026; baseline (n = 15) vs 3 h (n = 3): *p* = 0.0479, F(3,20) = 6.936, One-way ANOVA with Dunnett’s post-hoc test). ns-non-significant; *: *p* < 0.05; **: *p* < 0.01. All the data presented as mean ± SEM

The estimation of distances between the connected pairs of neurons can provide a piece of evidence about the spatial arrangement of neuronal pairs in the neuronal network. To compare the distribution of the dorsal hippocampal neuronal pairs in space, average distances between them were analyzed in both normal conditions and after acute stress. For the complete analysis of distance-related metrics, Euclidian distance (the direct distance between pairs of co-active neurons) and radial distance (the difference in distances between pairs of neurons from the center of all neurons’ mass) were used. To investigate the redistribution among neuronal circuits of the correlated neuronal pairs with relatively low (Fig. 5a, b) and high (Fig. 5c, d) correlation values, the mean value of both distances was calculated for normal and shifted states.

In the beginning, we compare the stability of distances between strongly correlated cell pairs in the normal condition for all mice (Figure S6). Euclidian and radial distance metric assumed to be stable if not more than 1 day of recording had significant differences in comparison to the others. Being grouped in this way, in the 83% of recordings distance values were stable during the baseline days of recordings. It was determined, that weakly correlated neuronal pairs displayed no significant redistribution in the conditions following acute stress modeling, maintaining stable mean values for both Euclidean distance (*p* = 0.9040, F(3,20) = 0.1869, One-way ANOVA test) and radial distance (*p* = 0.2875, F(3,20) = 1.347, One-way ANOVA test) (Fig. 5a, b). An opposite tendency was observed in strongly correlated neuronal pairs (Fig. 5c, d).

Following external exposure, the distances between correlated neuronal pairs with Pearson’s coefficient above 0.3 are significantly increased compared to baseline value. The most substantial differences were observed in the “stress” condition for Euclidian (baseline (n = 15) vs “stress” (n = 3): *p* = 0.0190, F(3,20) = 4.178, One-way ANOVA with Dunnett’s post-hoc test) and for radial (baseline (n = 15) vs stress (n = 3): *p* = 0.0026, F(3,20) = 6.936, One-way ANOVA with Dunnett’s post-hoc test). Furthermore, an elevation of the radial distance between co-active pairs of neurons is also observed in the “3 h” time-point (baseline (n = 15) vs “3 h” (n = 3): *p* = 0.0479, F(3,20) = 6.936, One-way ANOVA with Dunn’s post-hoc test). This strong reorganization, in particular, in the “stress” condition (146.9 ± 9.7 for baseline and 263.5 ± 49.3 for “stress” (Euclidian distance) and 57.05 ± 3.7 for baseline and 142.6 ± 30.4 for “stress” (radial distance)) among strongly correlated neurons may indicate different functions compared to weakly correlated ones. Moreover, this elevation was entirely reversed 10 days after exposure, suggesting that this spatial redistribution in the neuronal network space may reflect synaptic plasticity changes. We have also conducted normalized per-mouse analysis of correlated neurons distances (Fig. S7), revealing the same significant differences between states. Such changes, driven by great external stimulus, could reorganize the overall structure of the neuronal network to form highly correlated internal circuits for subsequent extraction [39, 40].

### Acute stress immobilization modeling lead to total neuronal shift within hippocampal neuronal ensembles

**Fig. 6:**
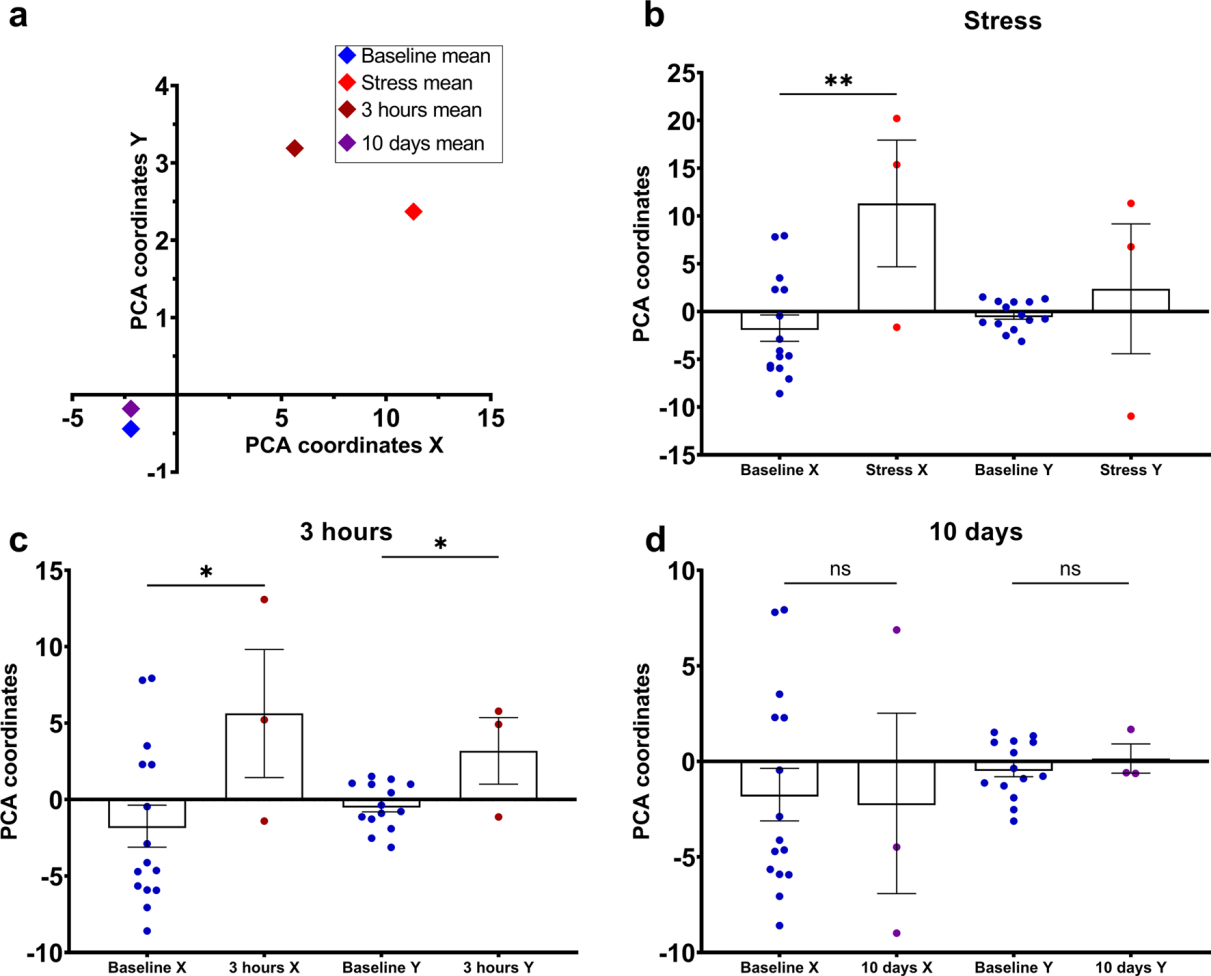
Hippocampal neuronal ensembles properties representation in 2D coordinates in normal conditions and at different time points after acute stress. **a** Total representation of the neuronal ensembles state in different experimental time points. PCA coordinates for baseline and **b** right after acute stress state (baseline (n = 15) vs stress (n = 3): X: *p* = 0.0054; Y: *p* = 0.3495, Student’s t-test). **c** 3 h post acute stress modeling (baseline (n = 15) vs 3 h (n = 3): X: *p* = 0.0447; Y: *p* = 0.0139, Student’s t-test for both). **d** 10 days after stress modeling (baseline (n = 15) vs 10 days (n = 3): X: *p* = 0.9020; Y: *p* = 0.5656, Student’s t-test). ns-non-significant; *: *p* < 0.05; **: *p* < 0.01. All the data presented as mean ± SEM

Great external exposure had a pronounced impact on various neuronal hippocampal ensembles characteristics. To validate the overall influence of this exposure on the entire neuronal net state and to gauge its stability through relevant characteristics, principal component analysis was implemented [41] (Fig. 6a). This approach was applied to the metrics, previously calculated for quantitative analysis of neuronal activity, as detailed earlier in the manuscript. The results are presented in Fig. 6.

As anticipated based on our data, the overall state of the neuronal circuits shifted away from their baseline level after acute stress (see Fig. 6b, c). The greatest affected metrics by external exposure were burst rate (i.e., number of activations per minute) and network spike peak (representing the peak number of simultaneously active neurons per 1 s) (refer to Fig. S8(a)). This correlates to observed dynamics of the metrics, connected with total neuronal activity, that shifted towards higher values while comparing with baseline level (Figs. 2d and 3). In contrast, metrics related to pairwise correlation calculations exhibited relatively minimal changes and demonstrated a higher degree of stability, seemingly resistant to alteration induced by the applied stimulus (Fig. S8). However, when examining the state of the hippocampal neuronal circuits network in the “10 days” time-point, no significant differences were observed (Fig. 6d).

## Discussion

Recording the activity of specific brain regions in vivo is essential for understanding their neuronal network structure and connectivity in physiological conditions. Analyzing patterns of neuronal activation within areas of interest with single-cell resolution can be a valuable tool for investigating normal and abnormal brain function [42–44]. Miniature fluorescent microscopy allows neuroscientists to record the activity of hundreds of neurons simultaneously in freely moving mice. In this study, we used the miniscope technique to examine the stability of hippocampal neuronal circuits under acute stress-induced conditions as a strong external stimulus for altering the network from its initial homeostatic state. Data on neural activity over a 5-day period in control conditions revealed a highly stable profile of neuronal activation at the single-cell level, as well as the distances between strongly connected pairs of neurons. Exposure to acute stress led to an immediate response from the hippocampal neuronal ensemble, resulting in an increase in several metrics, such as burst rate, network spike rate, and network spike duration. These metrics are closely linked to the activation profile of individual neurons within the neuronal circuits. At the same time, the connections between neurons, expressed as correlation coefficients, remained stable and were not affected by activation shifts in correlated pairs of neurons. However, there was a significant increase in the number of co-active neuronal pairs with weaker connections. Both of these results indicate a reorganization of the pairwise connections between neurons in response to a strong external stimulus. Specifically, the number of weakly correlated pairs increased, while the distance between these pairs remained unchanged. In contrast, the number of highly correlated pairs did not change, but the distance between those pairs increased. This suggests that the functional organization of the neuronal network architecture changes in response to changes in activation patterns, with no overall change in the average correlation coefficient.

At the same time, the behavior of strongly correlated neuronal pairs showed the opposite trend: an increase in the distance between co-active units, but no change in their number. This may be a way for the neuronal network to fine-tune itself in response to different stimuli by changing the “mission” of individual neurons in the hippocampal network, which represents temporal and spatial changes in neural activity [36]. However, it is still unclear whether this reorganization, which corresponds to the process of adjusting to strong external stimuli, can be found in the hippocampus or other brain regions [45]. When examining the overall state of hippocampal neuronal activity using the PCA method, a global shift was evident in both the reactive state (right after acute stress) and 3 h later. These states were significantly different from the initial condition, indicating a significant difference between normal and disturbed states. Thus, this finding suggests that the hippocampal neuronal network has a remarkable degree of flexibility and adaptability. Its functioning can be quickly adjusted to the surrounding stimulus. These changes in neuronal activity can persist for hours, even with a stronger response than the initial one. They may be associated with to various intracellular processes within neuronal cells, such as protein synthesis [24, 33] or strengthening of the synaptic connections among hippocampal neurons [32]. This shift may indicate overall synaptic potentiation in response to strong external stimulation, leading to increased neuronal excitation, synaptic strength, and network reorganization, similar to the processes of long-term potentiation and memory formation [46–49].

Moreover, on day 10 after exposure, no significant changes in network characteristics were observed. Metrics describing neuronal activity, correlations, and distances between correlated pairs remained very similar to those recorded under normal conditions. This confirms the exceptional stability of the hippocampal neuronal circuitry. Importantly, PCA analysis also showed a high degree of similarity between the neuronal ensemble representation under normal conditions and after 10 days, despite the significant changes observed during the stressful periods. Some subtle traces of the initial exposure may be present in other descriptors of the neuronal network, but all observed metrics did not change at all from baseline levels. In total, the hippocampal network has returned to its “home” state and no further changes can be detected. Various compensation mechanisms have been activated to stabilize the hippocampal network [49–52]. Thus, hippocampal neuronal ensembles exhibit high stability, allowing for consistent representation even after a significant external exposure, such as acute stress immobilization. These findings can provide new evidence for the concept of homeostatic synaptic plasticity in normally functioning brain areas [53–55]. However, whether the duration of acute stress exposure or other external stimuli correlates with the overall stability of hippocampal neuronal ensembles and the subsequent conversion to a “domestic state” is still unknown. Understanding the mechanisms underlying this possible transition and their manifestation in metrics describing neuronal networks is an urgent task. In summary, all data provides new evidence about the hippocampal neuronal circuits immediate response to a major external stimulus and its return to a “homeostatic” state.

## Materials and methods

### Animals

The breeding colony of C57BL/6J mice obtained from the Jackson Laboratory was established and maintained in a vivarium with 2–3 mice per cage and a 12 h light/dark cycle in the animal facility. Mice were kept in the vivarium of the Laboratory of Molecular Neurodegeneration of Peter the Great St. Petersburg Polytechnic University with a 12-h light cycle with ad libitum access to food and water. All efforts were made to minimize the number of animals used and their suffering. All procedures were approved by principles of the European convention (Strasburg, 1986) and the Declaration of International Medical Association regarding the humane treatment of animals (Helsinki, 1996) and approved by the Bioethics Committee of the Peter the Great St. Petersburg Polytechnic University (Ethical permit number 3-n-b from 25 May 2022) at St. Petersburg, Russia.

### Viral constructs delivery and GRIN-lens implantation

Implantation of GRIN-lens was performed in two-stages. Firstly, injections of viral constructs were done using a stereotaxic surgery (#68001, RWD Life Science, Guangdong, China), a syringe with a thin needle (#84853, 7758-02, Hamilton, Reno, NV, USA) and a heated mat with a temperature controller (69,002, RWD Life Science, Guangdong, China). Mice were anesthetized by gas mixture 1.5–2.0% of isoflurane. The depth of anesthesia was checked during the whole surgery by absence of pain stimulus to a single paw pinch. Injections of pAAV5.Syn.GCaMP6f.WPRE.SV40 at the titer of more than 1 × 10^13^ vg/mL were done under standard protocol [56] with following coordinates (AP −2.1; DV −1.8; ML +2.1) into left hemisphere. Volume of virus equaled to 1.4 µl at the rate of 0.1 µl per minute.

After 3 weeks of viral expression of calcium indicator GCaMP6f in the hippocampal neurons implantation of gradient lens was applied. Head of a mouse was fixed in the stereotaxic device and the skin was removed in the way for skull free access. Then, the skull was disinfected via 3% hydrogen peroxide. After a hole of 2 mm in diameter in the skull was drilled (Strong 90n, SAESHIN PRECISION CO, Daegu, Korea). Then the cortex and corpus callosum was aspirated until vertical fibers of corpus callosum can be visualized. All aspirations were done with continuous supply of sterile saline. After bleeding stopped, the gradient lens (#64519, Edmund Optics, Barrington, NJ, USA) was carefully and slowly lowered on the depth of 1.45 mm from the medial edge of the hole. Before fixation, all the skull was carefully scratched by drill machine without occurring any bleeding. Then, GRIN-lens was fixed to the skull with small volume of glue. Next, tiny screw was attached into skull-bone of the opposite hemisphere (all the GRIN-lens implantation were performed on the left-hemisphere of the mice). Surface of bone skull was covered via light-curing cement. During surgery sterile saline was applied at volume of 0.5 ml subcutaneously if needed. At the end of lens implantation 50 µl of atipam was injected intraperitoneal and 1 mg/kg of dexamethasone. Recovery after GRIN-lens implantation took approximately 4–6 weeks.

Afterwards, baseplate was fixed on the head with the best ROI of hippocampal neurons expressing GCaMP6f. Best field of view was detected by the highest amount of visible neurons either by clearly distinguishable blood vessels. Then, baseplate for further miniscope v3 fixation was fixed to the skull of the mouse by liquid-flowing composite material of light curing (Dent-light flow, tdVladmiva, Russia). Following recovery from baseplate implantation (about 3–4 weeks) and isoflurane exposure mice were in vivo imaged by miniscope v3 (Labmaker, Berlin, Germany).

### Hippocampal neuronal activity recordings of freely behavior mice

After total recovery of male mice aged 8 months old (n = 3), they were allowed to habituate to miniscope fixed on their head for 7 min once a day 3 days long in the experimental environment. Experiments of neuronal activity recording in the freely moving mice were performed in the Open Field chamber. It was rounded arena made of opaque plexiglass with a diameter of 63 cm. Miniscope was fixed on baseplate before recordings without isoflurane or another anesthesia for obtaining the most biological signal, because anesthesia strongly suppresses neuronal activity and GCaMP6f fluorescence intensity. Base level of the neuronal network of hippocampus activity was recorded every day for 7 min under the same conditions for 5 consecutive days without any external influence on the mice behavior. It should be noted that imaging experiments were conducted approximately 4 months after the expression of GCaMP6f in the mice hippocampus. This may have led to some limitations, such as for example saturation of the calcium indicator’s signal. After each recording ended, the chamber was sterilized with 70% ethanol and after drying the next mouse was recorded. When the initial baseline level of hippocampal neuronal ensembles activity within 5 days was obtained, acute stress immobilization was applied to mice. Mice were physically restrained in well-ventilated 50 ml Falcon tubes (91050, TRP, Trasadingen, Switzerland) for 30 min. Right after recording of neuronal activity in the open field chamber were performed, then after 3 h of acute stress and 10 days after in the normal conditions in the same Open Field chamber. Data from 3 mice was analyzed (mean amount of recorded neurons for analyzed mice: 171.2 ± 15.4). So baseline level, states after the acute restrained test of “stress”, “3 h” and “10 days” characterize 3 mice hippocampal neuronal ensembles.

### Processing of miniscope recordings

Miniscope data was recorded via free access Portable Miniscope Data Acquisition program (Pomidaq) at 15 frames per second rate. For each mouse excitation parameters were individually found but never excessed 50% of maximum intensity value when gain value for all mice were set at “medium” in Pomidaq (v. 0.4.5). All the recordings were obtained in the “.mkv” format. Each recording was cut into 5-min-long duration fragments so the first and the last minute were discarded from analysis. After formatting it into “.avi” the next step of processing was performed—processing miniscope data using Minian [57]. It is an open source product for processing of miniscope data. It allows to perform fluctuating background elimination, motion correction and calcium data extraction via CNMF method [58]. For processing of miniscope data following characteristics of variable characteristics in Minian were used: “wnd_size”: 1000, “method”: “rolling”, “stp_size”: 500, “max_wnd”: 15, “diff_thres”: 3 for initialization parameters and with standard parameters for CNMF method inside Minian.

Minian gives an array with information about neuronal calcium activity (Ca^2+^ fluorescence traces) and location of each neuron in the recording. For the following quantitative analysis of the miniscope data special toolbox NeuroActivityToolkit [32].

### Quantitative analysis of miniscope recordings

All the preprocessed recordings via Minian pipeline were analyzed by NeuroActivityToolkit [32]. It is open-source toolbox for quantitative analysis of miniscope recorded data. First, active states of neurons were validated by “spike” method as a phase of rapid growth of calcium indicator fluorescence intensity with next parameters: “cold” value equaled 0 (the minimum duration of the active phase), “warm” value equaled 50 (the minimum duration of the passive phase) and “window” size equaled 10 (frames value for smoothing initial calcium trace) that are counted in frames. All the calculated statistics, presented in the current manuscript, are based on the active state determining by “spike” method if other non-mentioned. For correlation analysis on the Fig. 4 “full” method was also used. “Full” method highlighted part of the calcium trace which is higher than computed threshold value and is less strict way for detection of the neuronal active state. Moreover, for PCA method all the metrics are calculated by “spike”, “full”, “signal” and “diff” methods. “Signal” is a method for active state validation where only intensity of the original fluorescence is taken into account. It is the less strict way for metrics computing. “Diff” method determine active state of the neuron as derivative of its fluorescence intensity. For principal component analysis also “active accuracy” method was implemented to find correlation between intersection to the union of active states of neurons.

“Burst rate” statistical metric describes amount of neuronal activation per minute in the preset intervals. “Network spike rate” is a percentage of active neurons per set interval of time. “Network spike peak” is maximum amount of the simultaneously active neurons. For calculations of these two metrics interval was set as 1 s. “Network spike duration” is a value of simultaneously active neurons above threshold which were set as 2.5, 5.0, 10.0, 15.0, 20.0, 25.0%. For distance analysis Euclidian (direct distance between co-active neuronal pairs) and radial (difference in distances between pairs of neurons from the mass center of all neurons in the recording) distances were used. For PCA method all the possible metrics and their variation were used. In Figure S4 also some earlier not mentioned metrics could be found: “connectivity”—percent of connections with other neurons for each neuron, “intercluster distance” is mean value of correlation coefficient for neurons with out-of-cluster cells and “intracluster distance” is mean value of correlation coefficient for individual highly correlated neuronal group (pair of neurons belongs to the same cluster if the correlation values between them are stronger than 80% of the connections in the network).

### Tracking the same neurons activity across sessions

To track the same neurons across days in miniscope recordings, we implemented the CellReg method [34]. A parameter of 7 pixels was used to identify neurons as the same cell across sessions. To account for natural variability in the neurons activity through base level days of recording, we have normalized each corresponding neurons to their activity on the day 5. To determine threshold levels for subsequent analysis, we have found median values of normalized activation per minute values at baseline days. At the base level stage, median level of normalized activations per minute for “activation” equaled 1.75 and for “inhibiting” it valued 0.5. Thus, these parameters were used to identify neurons to be activated (normalized activations per minute >1.75), non-responded (0.5< normalized activations per minute <1.75) or inhibited (normalized activations per minute <0.5) by strong external stimuli.

### Fixed-slice preparation

For fixed-slice preparation mice were firstly anesthetized by urethane (150 mg/ml) and rometar (32 µl/ml) diluted in 0.9% NaCl. After the onset of the effect of anesthesia, a needle was inserted into the left ventricle, through which a fixing solution was pumped. Fixing solution was 1.5% PFA (pH = 7.4) solution in PBS. The duration of perfusion was 5–7 min, after which the brain was decapitated and extracted, followed by its storage in a paraformaldehyde solution for a week. 50 µm slices were obtained via Campden 5100 mz Microtome (Campden Instruments vibrating microtome Ltd, Loughborough, England). Slices were kept in 0.5% PFA. For microscopy slices were glued with Aqua Poly/Mount (Polysciences, #18606, Warrington, PA, USA) on microscope slides (Heinz Herenz, # 1042050, Hamburg, Germany) and coverslips (Menzel Glaser, #1, Germany). Light microscopy images were made by means of OLYMPUS IX73 microscope (OLYMPUS, Tokyo, Japan) and UPlanFL N 10× objective (OLYMPUS, Tokyo, Japan). Confocal images of GCaMP6f fluorescence in the hippocampal neurons, presented on Fig. 1b were made by Leica TCS SP8 microscope.

### Statistics

Firstly, all the data samples of metrics, describing neuronal properties of the hippocampal network, were analyzed on normality via Shapiro–Wilk or Kolmogorov–Smirnov test. Further, for normally distributed samples Barllet’s test was applied to ensure of homogeneity of the values. Before statistical analysis, we have checked all the data on outliers. Based on the obtained test results data was compared by Student t-test or Mann–Whitney test for paired comparison and via ANOVA test following Tukey, Dunnett’s, Fisher’s LSD test or Kruskal–Wallis test following by Dunn’s test for multiple comparisons. Statistically significant differences were considered with *p* < 0.05. All the data presented in the pictures and in text are shown as mean ± standard error of the mean.

## Supplementary Information

Additional file 1.

## Data Availability

All the data (raw videos and preprocessed data) are available upon reasonable request to corresponding authors. Quantitative analysis of miniscope preprocessed data was done via self-made open-source toolbox that could be found here: https://github.com/spbstu-applied-math/NeuroActivityToolkit.
