# Supplementary material for: Dynamic changes in the hippocampal neuronal circuits activity following acute stress revealed by miniature fluorescence microscopy imaging
Source: Mol Brain. 2024 Dec 18;17:92. doi: 10.1186/s13041-024-01168-5 (PMC11653891; doi:10.1186/s13041-024-01168-5)
Supplement: Supplementary file 1 — Additional file 1. [file 13041_2024_1168_MOESM1_ESM.docx]

**Supplementary material**

**
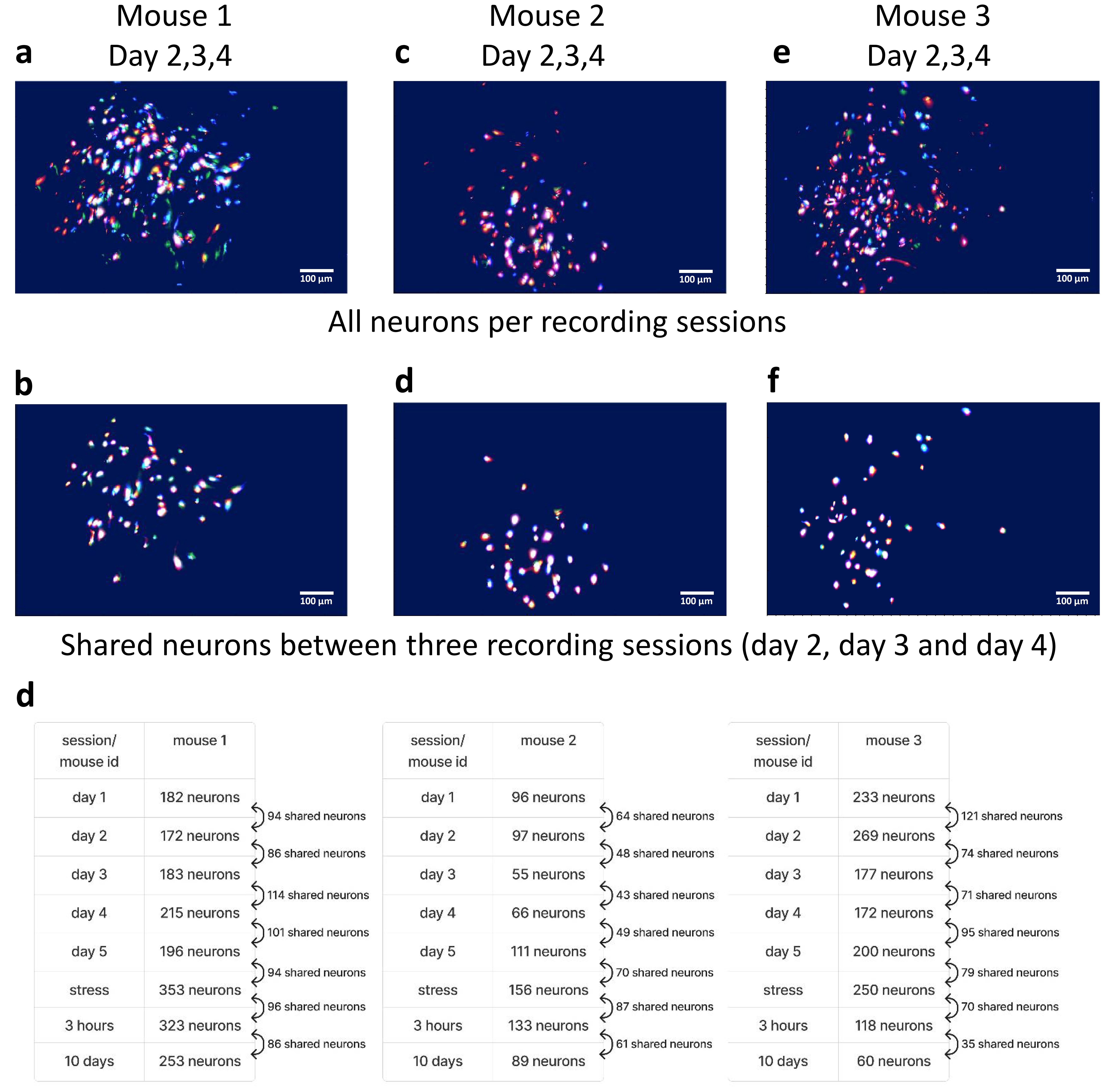
**

**Figure S1**. **Imaging the same hippocampal neuronal ensembles *in vivo* using miniscope in the freely moving mice. (a), (c), (e)** Recorded neurons from hippocampus in the freely moving sessions at day 2,3 and 4. Neurons are labeled as: day 2 – red color, day 3 – green color, day 4 – blue color. Intersected neurons are visualized in white. **(b), (d), (f)** Shared neurons across 3 consecutive days of recording. **(d)** Amount of detected neurons in all recording sessions. Double-side arrow indicates number of shared neurons between recordings.

**

Figure S2**. **Increase in the neuronal activations number in binarized form after acute stress modeling as great external perturbation.** White lines indicate single neuron activation.

**
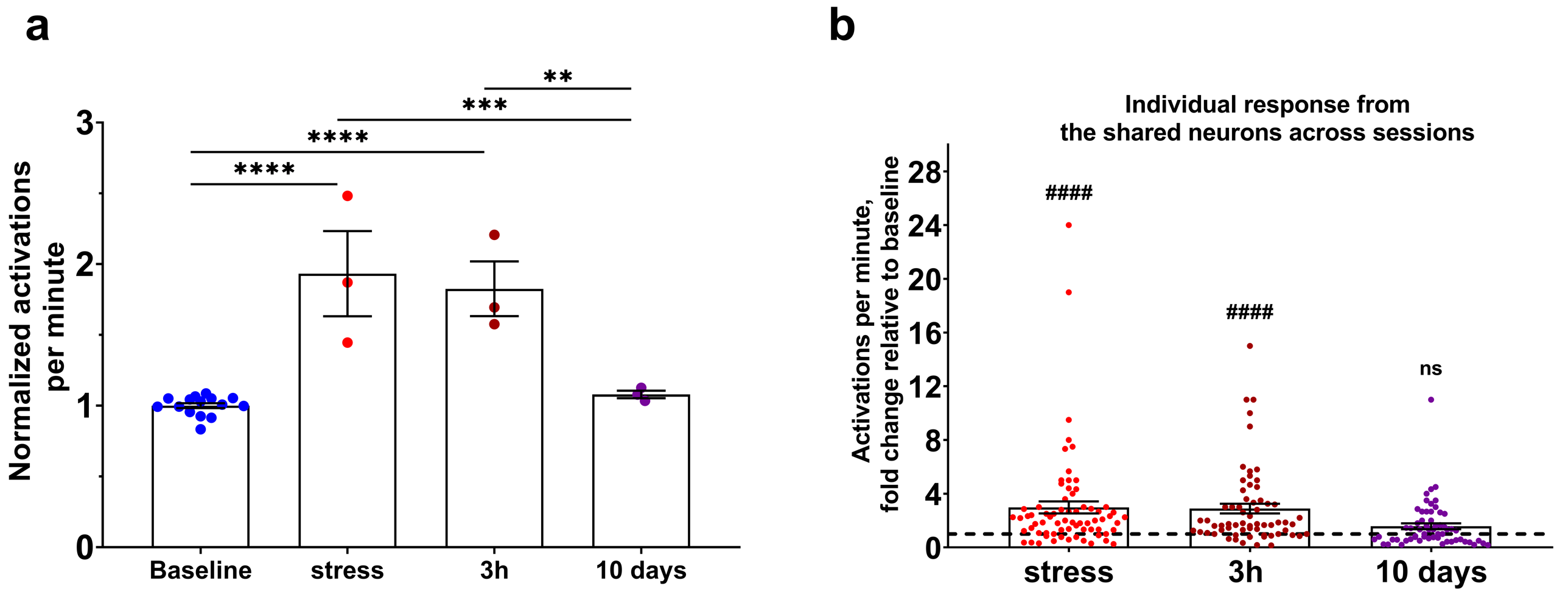
**

**Figure S3**. **Normalized values of calcium events and individual response from the shared neurons between sessions for all states.** (**a**) Baseline (n=15) vs stress (n=3), p<0.0001; baseline (n=15) vs 3 hours (n=3), p<0.0001; baseline (n=15) vs 10 days (n=3), p=0.9265; stress (n=3) vs 3 hours (n=3), p=0.9175; stress (n=3) vs 10 days (n=3), p=0.0003; 3 hours (n=3) vs 10 days (n=3), p=0.0012; Ordinary One-way ANOVA with Tukey test for multiple comparison, F(3,20)=27.13. (**b**) Individual response from the shared neurons between sessions for all states. Comparison for each neurons to its baseline, baseline (day 5) (n=73 neurons) vs stress (n=73 neurons), p<0.0001; baseline (day 5) (n=73 neurons) vs 3 hours (n=64 neurons), p<0.0001; baseline (day 5) (n=73 neurons) vs 10 days (n=59 neurons), p=0.6251; Kruskal-Wallis test following Dunn’s test for multiple comparisons. Baseline level is depicted with dotted line and equaled 1, as each neuron’s activity was normalized to the same correspondent neuron baseline activity. ns: no significant difference, *: p<0.05; **: p<0.01; ***: p<0.001; ****: p<0.0001; ^####^: p<0.0001. All the data presented as mean ± SEM.

**

**

**Figure S4**. **Multiple comparison of metrics characterizes total activity of the hippocampal neuronal circuits.** (**a**) Burst rate distribution (calcium transients number to their relative amount) comparison with baseline level across different states (baseline (n=15) vs stress (n=3): *: p <0.05, **: p <0.01; baseline (n=15) vs 3 hours (n=3): #: p <0.05; baseline (n=15) vs 10 days (n=3): ⊥: p<0.05, Kruskal–Wallis test with multiple comparisons by Dunn’s test). **(b)** Network spike rate distribution (percent of neurons in active state in the distinct interval of time) comparison with baseline level across different states (baseline (n=15) vs stress (n=3): *: p <0.05, **: p <0.01; baseline (n=15) vs 3 hours (n=3): #: p <0.05, ##: p <0.01. Kruskal–Wallis test with multiple comparisons by Dunn’s test. **(c)** Network spike duration (the time during which the count of concurrently active cells exceeds a predetermined threshold level) comparison with baseline level across different states (baseline (n=15) vs stress (n=3): *: p <0.05, **: p <0.01; baseline (n=15) vs 3 hours (n=3): #: p <0.05, Kruskal–Wallis test with multiple comparisons by Dunn’s test). **(d)** Network degree distribution (the amount of co-active pairs above predetermined threshold level) comparison with baseline level across different states (baseline (n=15) vs stress (n=3): *: p <0.05, **: p <0.01; baseline (n=15) vs 3 hours (n=3): #: p <0.05, Ordinary one-way ANOVA analysis with multiple comparisons by Fisher’s LSD test). All the data is presented as mean ± SEM.





**Figure S5. Frequency distributions for all experimental states conditions.** Cumulative probability comparison for neuronal activations per minute between **(a)** baseline (n=2344 neurons from 15 sessions (N=3 mice)) vs stress (n=754 neurons (N=3 mice)): p=0.0011. **(b)** baseline (n=2344 neurons from 15 sessions (N=3 mice)) vs 3 hours (n=564 neurons (N=3 mice)): p=0.0165. **(c)** baseline (n=2344 neurons from 15 sessions (N=3 mice)) vs 10 days (n=386 neurons (N=3 mice)): p>0.9999. Cumulative probability comparison for active neurons per second between **(d)** baseline (n=4312 time sections from 15 sessions (N=3 mice)) vs stress (n=877 time sections (N=3 mice)): p <0.0001. **(e)** baseline (n=4312 time sections from 15 sessions (N=3 mice)) vs 3 hours (n=865 time sections (N=3 mice)): p=0.0381. **(f)** baseline (n=4312 time sections from 15 sessions (N=3 mice)) vs 3 hours (n=867 time sections (N=3 mice)): p=0.9685.


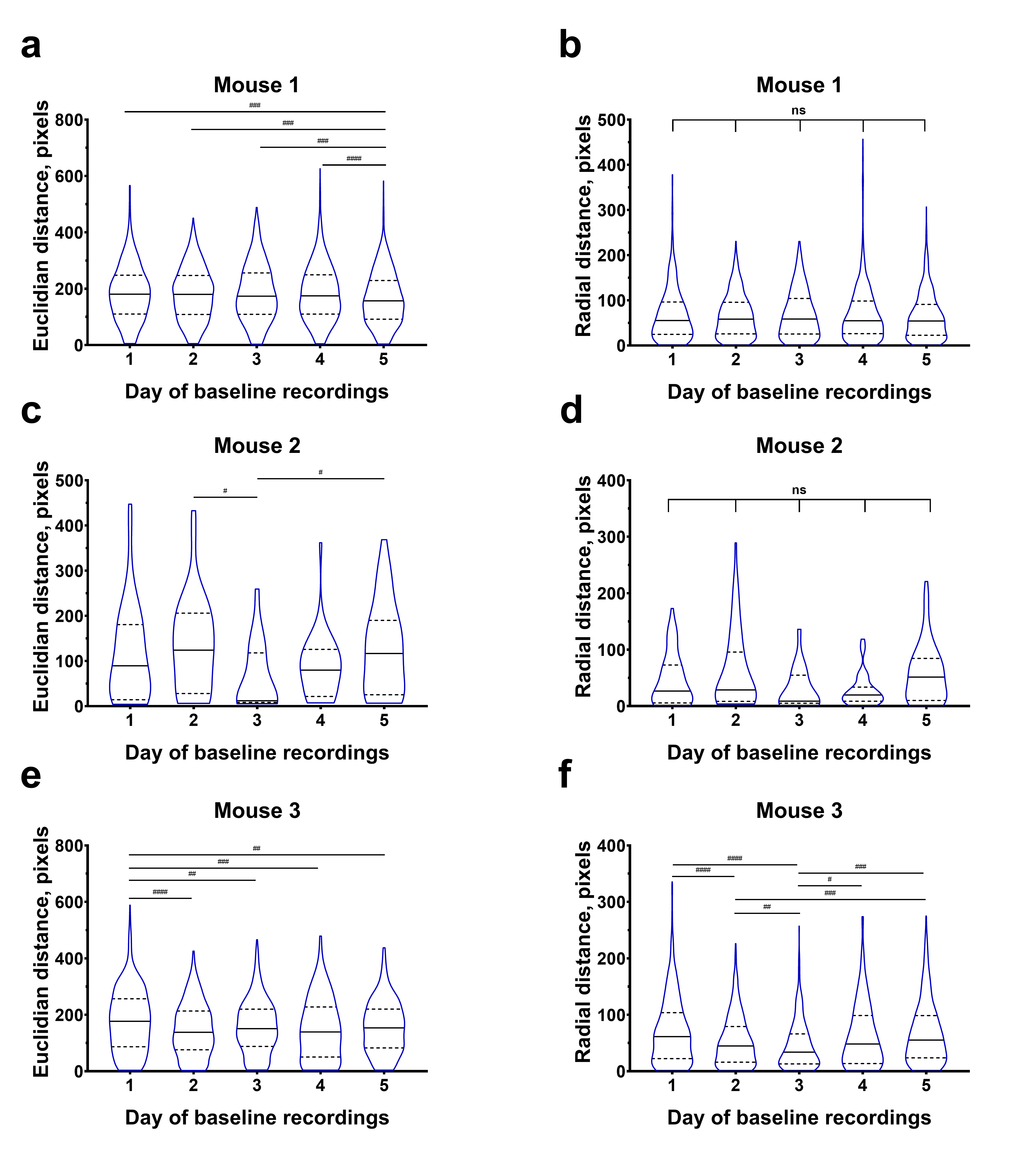


**Figure S6**. **Stability of the Euclidian and radial distances between strongly correlated cell pairs (Pearson’s correlation coefficient > 0,3) in the normal condition for individual mice.** **(a)** Euclidian distance between strongly correlated pairs of neurons (1day vs 5day: p=0,0003; 2day vs 5day: p<0,0001; 3day vs 5day: p=0,0004; 4day vs 5day: p<0,0001) for mouse 1. **(b)** Radial distance between strongly correlated pairs of neurons for mouse 1. **(c)** Euclidian distance between strongly correlated pairs of neurons (2day vs 3day: p=0,0223; 3day vs 5day: p=0,0200) for mouse 2. **(d)** Radial distance between strongly correlated pairs of neurons for mouse 2. **(e)** Euclidian distance between strongly correlated pairs of neurons (1day vs 2day: p <0,0001; 1day vs 3day: p=0,0085; 1day vs 4day: p=0,0006; 1day vs 5day: p=0,0095) for mouse 3. **(f)** Radial distance between strongly correlated pairs of neurons (1day vs 2day: p<0,0001; 1day vs 3day: p<0,0001; 2day vs 3day: p=0,0303; 2day vs 5day: p=0,0002; 3day vs 4day: p=0,0138; 3day vs 5day: p<0,0001) for mouse 3. All the comparisons are performed via Kruskal-Wallis test with following Dunn’s test for multiple comparisons. All the data presented and violin plots with median (bold line) and first and third quartiles (top and bottom dotted lines correspondingly).


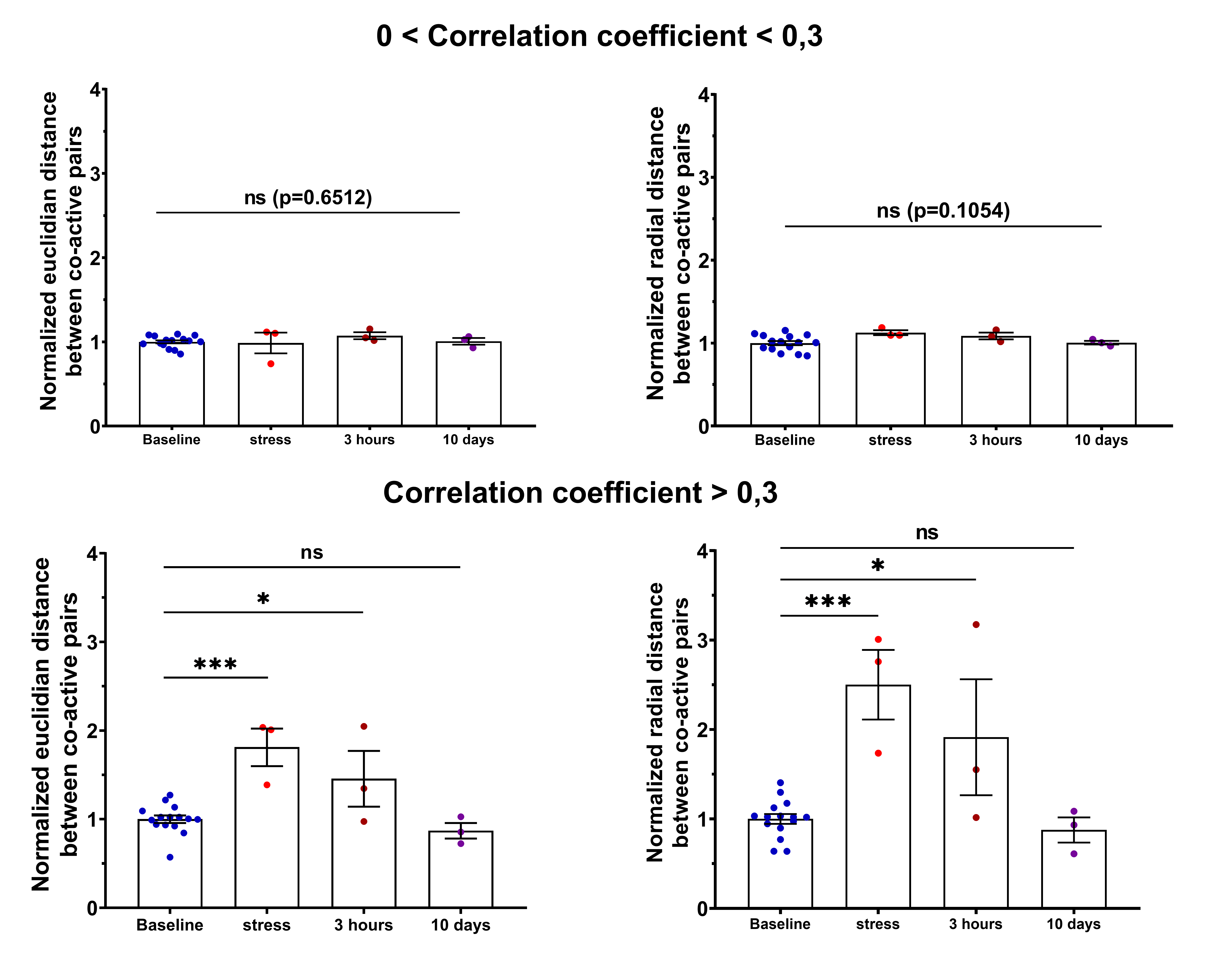


**Figure S7**. **Normalized distances between correlated hippocampal neuronal pairs in space in a response to acute stress.** **(a)** Normalized Euclidian distance for weakly correlated neurons (p=0.6512, F(3,20)=0.5542, One-way ANOVA test). **(b)** Normalized radial distance for weakly correlated neurons (p=0.1054, F(3,20)=2.328, One-way ANOVA test). **(c)** Normalized Euclidian distance for strongly correlated neurons (baseline (n=15) vs stress (n=3): p=0.0002; baseline (n=15) vs 3 hours (n=3): p=0.0284, F(3,20)=11.28, One-way ANOVA with Dunnett’s post-hoc test). **(d)** Normalized radial distance between strongly correlated neuronal pairs (baseline (n=15) vs stress (n=3): p=0.0001; baseline (n=15) vs 3 hours (n=3): p=0.0144, F(3,20)=11.70, One-way ANOVA with Dunnett’s post-hoc test). ns-non-significant; *: p<0.05; **: p<0.01. All the data presented as mean ± SEM.


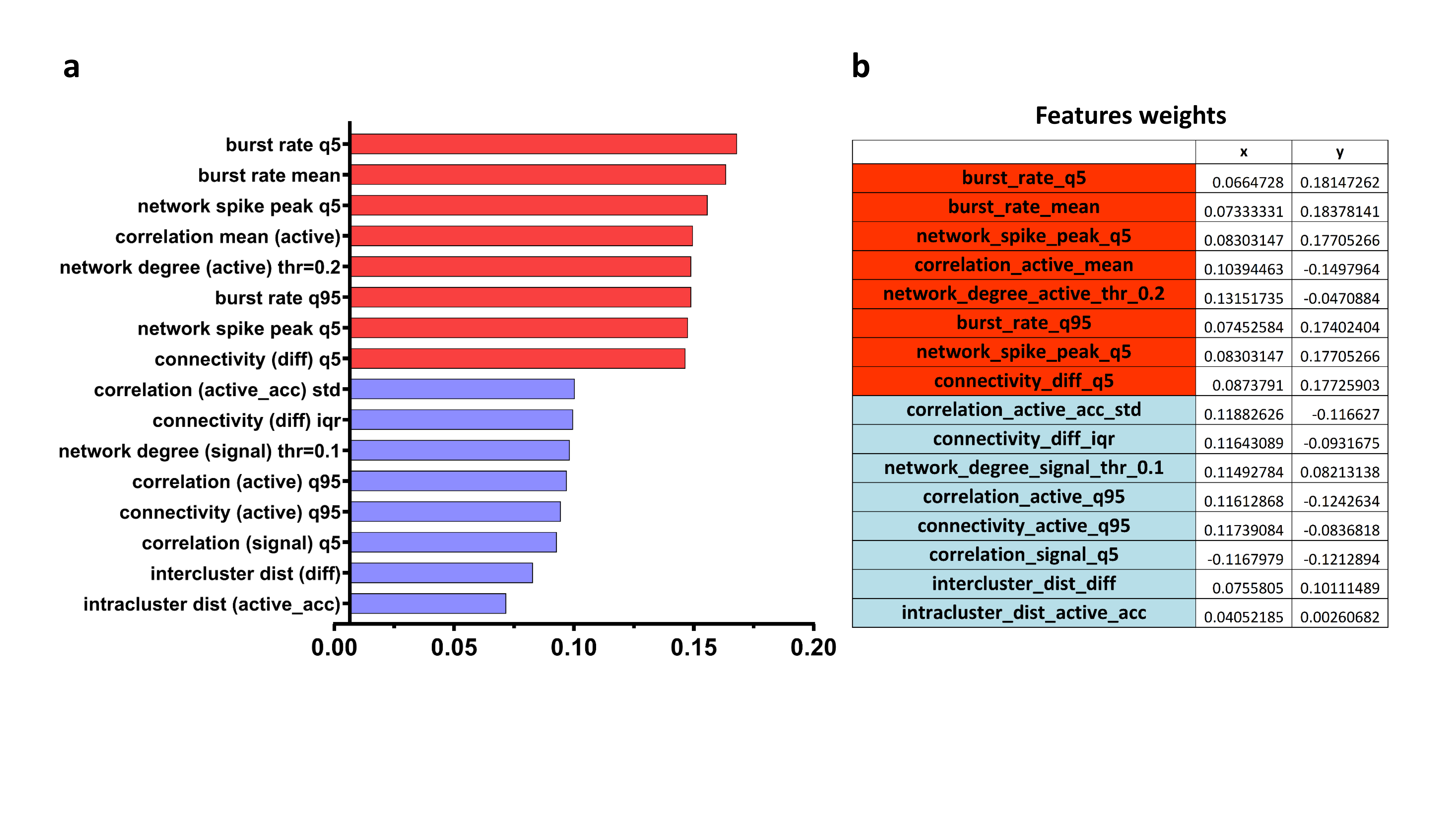
**Figure S8. The highest and the lowest stable metrics, characterizing hippocampal neuronal network.** **(a)** Mean absolute deviation of the metrics. **(b)** Feature weights of the most and least stable descriptors for PCA method. By red color highlighted the most changeable characteristics and by blue color the most stable. q5- lowest 5 percent of values; q95 – highest 5 percent of values; std – standard deviation; iqr – interquartile range as difference between Q1 and Q3; thr – threshold value.
